# Potential Susceptibility Mutations in *C* Gene for Hepatitis B-Related Hepatocellular Carcinoma Identified by a Two-Stage Study in Qidong, China

**DOI:** 10.3390/ijms17101708

**Published:** 2016-10-11

**Authors:** Lishuai Qu, Haifeng Zhang, Jinxia Liu, Taotao Liu, Xizhong Shen, Taoyang Chen, Zhengpin Ni, Cuihua Lu

**Affiliations:** 1 Department of Gastroenterology, Affiliated Hospital of Nantong University, Nantong 226001, China; qulishuai1121@163.com (L.Q.); liujinxia850213@163.com (J.L.); 2 Department of Infectious Disease, Affiliated Hospital of Nantong University, Nantong 226001, China; firend20942@163.com; 3 Department of Gastroenterology, Zhongshan Hospital, Fudan University, Shanghai 200032, China; liutaotao3@yeah.net (T.L.); shenxizhong@126.com (X.S.); 4 Qidong Liver Cancer Institute, Qidong 226200, China; chentaoyang2@yeah.net (T.C.); nizhengpin@yeah.net (Z.N.)

**Keywords:** hepatocellular carcinoma, mutation, C gene, hepatitis B virus

## Abstract

A two stage study was conducted to explore new potential mutations in the full genome of hepatitis B virus (HBV) on the progression of hepatocellular carcinoma (HCC) in Qidong, China. In stage 1, full genomes of HBV were compared between 30 HCC cases and 30 controls. In stage 2, an independent case–control study including 100 HCC cases and 100 controls was enrolled to verify the relationship between hot-spot mutations and HCC development. Furthermore, a longitudinal study was conducted on 11 HCC cases with serial serum samples available before HCC diagnosis. A total of 10 mutations (including pre-*S*2 start codon mutation and pre-*S* deletion in pre-*S* gene, G1613A, C1653T, A1762T, and G1764A mutations in *X* gene, A2159G, A2189Y, G2203W, and C2288R mutations in *C* gene) showed an increased risk of HCC. In the validation study, pre-*S* deletion, C1653T, A1762T/G1764A, A2159G, A2189Y, G2203W, and C2288R mutations were associated with increased HCC risk in univariate analysis. Multivariate analysis indicated that pre-*S* deletion, A1762T/G1764A, A2159G, and A2189Y mutations were independently related with HCC development. Moreover, a significant biological gradient of HCC risk by number of mutations in the *C* gene was observed. Longitudinal observation demonstrated a gradual combination of the above mutations accumulated during the progression of HCC.

## 1. Introduction

Hepatocellular carcinoma (HCC) is a global health problem, as it is the fifth most common cancer and the third leading cause of cancer-related death [1]. Etiologically, the majority of HCC develops in chronic hepatitis B virus (HBV) carriers, especially in East Asia and sub-Saharan Africa, where HBV is endemic [2]. Although an effective vaccine has been used for about two decades, more than 350 million people in the world are chronic carriers of this virus [3]. Until now, the exact mechanism underlying hepatocarcinogenesis in chronic HBV infection remains elusive.

The HBV genome comprises a partially double-stranded circular DNA molecule of approximately 3200 base pairs. This DNA strand encodes four overlapping open reading frames (ORFs): *X*, for the *X* protein; precore/core (*C*), for the nucleocapsid; pre-*S*/*S*, for the surface/envelope protein; and *P*, for the DNA polymerase [4]. HBV replicates through an RNA intermediate, using a reverse transcriptase that lacks a proofreading function. Thus, HBV exhibits replication errors at a much higher rate than in other DNA viruses, and the estimated mutation rate is about 1 nucleotide/10,000 bases per year [5]. The effect of viral mutations on HCC pathogenesis has been investigated extensively, and several important high-risk mutations have been identified. The most convincing association between viral mutation and the development of HCC is A1762T/G1764A double mutations in the basal core promoter (BCP) [6,7]. Additionally, pre-*S* deletion, T1753V mutation in BCP, and C1653T mutation in box-α of Enhancer II have been reported to be related with increased risk of HCC in several reports [8,9,10,11,12]. However, current studies concerning other potential predictive mutations by comparative analysis of the complete HBV genomes remain limited. Meanwhile, distinct clinical and virologic characteristics of HBV infection have been reported in different geographical parts of the world, and are increasingly associated with genetic diversity of the infecting virus [13]. The township of Qidong is one of the highest endemic regions for HBV-related HCC in China. In this two-stage study, the complete HBV genome was initially analyzed in the serums of patients from a prospective cohort of male HBV carriers in Qidong, in order to explore new mutation biomarkers of HCC development in addition to traditional hot-spot mutations in pre-*S* and *X* genes. Second, an independent validation study was conducted to confirm the relationship between the newly identified mutations and HCC risk. Furthermore, sequential serum sequencings of the *C* gene were carried out to assess the longitudinal evolution of mutations in the C gene during HCC development.

## 2. Results

### 2.1. Comparison of Full Hepatitis B Virus (HBV) Genome between Hepatocellular Carcinoma (HCC) Cases and Controls

In stage 1, the full sequences of HBV in 30 HCC cases and 30 controls were determined by PCR direct sequencing. There were no statistically differences in age, distribution of genotype, and seropositivity of hepatitis B e antigen (HBeAg) between HCC cases and controls (55.0 ± 9.0 versus 56.3 ± 8.9 years, *p* = 0.874 for age; 7/30 (23.3%) versus 8/30 (26.7%), *p* = 0.766 for seropositivity of HBeAg; 2:28 versus 3:27, *p* = 0.640 for the genotype B to genotype C ratio). The number of nucleotide substitutions in the full genome was calculated by comparing with each corresponding prototype sequence from GenBank (Version GI289976889 for genotype C and GI289976881 for genotype B, both from an HBV carrier in Qidong, China). The average nucleotide substitutions in the full genome were 66.3 ± 10.4 and 57.6 ± 7.8 in HCC cases and controls (*p* = 0.001), respectively. Table 1 shows the number of nucleotide substitutions in various regions of the HBV genome. The HCC group had significantly more nucleotide substitutions in pre-*S*2 (*p* = 0.015), *X* (*p* = 0.002), and pre-*C*/*C* (*p* = 0.016) regions, respectively. The *P* gene indicated slightly increased nucleotide substitutions in HCC cases compared with controls (*p* = 0.112).

### 2.2. Association between HCC Risk and the Presence of Specific Mutations

Table 2 lists the frequencies of 24 hot-spot mutations observed in the full genome of HBV in HCC cases compared to controls. These include some well-studied mutations, such as pre-*S*2 start codon mutation, pre-*S* deletion, C1653T, T1753V, A1762T/G1764A, and G1896A mutations. A total of 10 mutations showed significant differences between HCC cases and controls. Among these, 10 were point mutations, two (pre-*S*2 start codon mutation and pre-*S* deletion) were located in the pre-*S* gene, four (G1613A, C1653T, A1762T, and G1764A mutations) were located in the *X* gene, and four (A2159G, A2189Y, G2203W, and C2288R mutations) were in the *C* gene. However, we did not observe even one point mutation in *S* and *P* genes that showed a significantly different frequency between HCC and control groups in this study. These data suggested that high HCC risk mutations were not likely to distribute evenly throughout the whole HBV sequence.

### 2.3. Validation of HCC-Related Hot Spot Mutations in the C Gene

In stage 2, to confirm the risk of 10 point mutations from stage 1 during the development of HCC, an independent case–control study was conducted with 100 HCC cases and 100 controls. After excluding the subjects with poor sequence data or negative PCR product (1 case and 2 controls), a total of 99 HCC cases and 98 controls were included in the final analysis. The demographic data of the 99 HCC patients and 98 controls are listed in Table 3. There were no statistically significant differences in age, history of cigarette smoking and alcohol consumption, seropositivity of HBeAg, or serum HBV DNA levels between HCC cases and controls. Genotype C dominated the HBV types in Qidong, accounting for 95.4% of HCC cases and 93.4% of controls. HCC cases and controls showed a similar distribution pattern for genotype (*p* > 0.05). When we examined HBV DNA sequences in the pre-*S* and Enh II/BCP regions, pre-*S* deletion, C1653T, and A1762T/G1764A, double mutations were significantly associated with HCC, showing adjusted ORs from 1.929 to 2.385 (Table 3). The most frequently occurring mutation was a T1762/A1764 double mutation. However, G1613A (OR, 1.769; 95% CI, 0.932–3.358) and pre-*S*2 start codon (OR, 2.233; 95% CI, 0.795–6.274) mutations were not related to a higher risk of developing HCC. Meanwhile, the frequencies of hot-spot mutations in the *C* gene increased in the HCC group, from 6.1% (deletion), 15.2% (T1938C), 11.1% (C2002T), 13.1% (T2045A), 35.4% (A2159G), 40.4% (A2189Y), 10.1% (G2203W), and 22.2% (C2288R) in HCC cases to 2.0%, 7.1%, 7.1%, 14.3%, 20.4%, 22.4%, 2.0%, and 11.2% in controls, respectively. Similar to the results from initial full genome analysis, HCC patients had significantly higher frequencies of A2159G, A2189Y, G2203W, and C2288R mutations than controls (*p* = 0.020, *p* = 0.006, *p* = 0.037, and *p* = 0.030, respectively). After adjustment for age, history of cigarette smoking, and alcohol consumption, unconditional logistic regression analyses showed adjusted ORs from 2.147 to 5.203 (Table 3). Therefore, using stepwise logistic regression analysis, the following were found to be independent risk factors of HCC: A2159G (OR, 2.037; 95% CI, 1.033–4.015), A2189Y (OR, 2.833; 95% CI, 1.453–5.526), pre-*S* deletion (OR, 2.272; 95% CI, 1.037–4.979), and A1762T/G1764A double mutations (OR, 2.333; 95% CI, 1.240–4.390) (Table 4).

### 2.4. Association between HCC Risk and the Presence of Specific Mutation Patterns in C Gene

In the current study, the risk of combined mutations in the *C* gene on HCC was explored, including the A2159G, A2189Y, G2203W, and C2288R mutations. The different patterns of combined mutations are presented in Table 5. Wild type and single mutations were highly prevalent, and were found in 40.6% and 41.1% of the included subjects, respectively. Double mutations were relatively rare and only occurred in 13.7% of the subjects, triple mutations were only found in 9 (4.6%) patients. Our data revealed that any mutation combinations were significantly associated with a higher risk of HCC. Compared to patients with wild-type infection, the adjusted OR was 2.904 (95% CI, 1.508–5.590) in those with a single mutation; 6.027 (95% CI, 2.232–16.275), double mutations; 8.630 (95% CI, 1.588–46.891), triple mutations. We did not observe any subject with quadruple mutations in these four points. A significant biological gradient of HCC risk by number of mutations in the *C* gene was observed.

### 2.5. Longitudinal Observation of C Gene Mutations during HCC Development

Most previous studies aiming to better understand the relationship between HBV mutation and HCC were conducted with a single blood sample at enrollment or upon HCC diagnosis. In this study, we further explored the HBV mutations in serial serum samples spanning years to assess the evolution of mutations in the *C* gene during HCC development. Analysis was focused on those putative HCC-related mutations identified from the above cross-sectional study. Among 99 HCC cases with success sequence data, 11 HCC cases with adequate sequential serum samples were selected for this longitudinal investigation of specific mutation patterns (A2159G, A2189Y, G2203W, and C2288R mutations). The lack of some information was due to a negative PCR product. Table 6 demonstrated the evolution of *C* gene mutations during the progression of HCC. Among these 11 HCC cases, seven cases had at least one nucleotide substitution of these four high HCC risk mutations at HCC diagnosis, and five cases of seven showed a gradual accumulation of these mutations in gene *C* during follow-up. Reverse mutation was observed in only one patient. These results, together with those from our case-control study, indicated that the high HCC risk mutations in the *C* gene were not acquired at the beginning of HBV infection, but occurred during the long course of liver disease.

## 3. Discussion

HBV infection is a major risk factor for HCC occurrence; ≥75% cases of HCC are associated with HBV infection in China [14]. Compared with non-carriers, patients with chronic HBV infection have a greater than 100-fold increased risk of developing HCC [15]. During the course of chronic HBV infection, a wide variety of liver diseases are observed, ranging from an asymptomatic carrier state to liver cirrhosis and HCC [16]. In our previous studies conducted in Qidong, pre-*S* deletion and specific mutations in BCP were confirmed to be associated with a high risk of HCC occurrence [17,18]. However, the risk of mutations in other regions of the full HBV genome was seldom reported. In the present study, the full HBV genome was analyzed in the serum of patients within a large cohort of male HBV carriers in Qidong. The number of nucleotide substitutions in the full-length sequence was significantly higher in HCC cases than in controls. Meanwhile, our data demonstrated that high HCC risk mutations were not likely to distribute evenly throughout the complete HBV genome. The regions with significant differences in the mutation number between HCC and control patients were (in rank order) *X* (*p* = 0.002), pre-*S*2 (*p* = 0.015), pre-*C*/*C* (*p* = 0.016), *P* (*p* = 0.112), pre-*S*1 (*p* = 0.483), and *S* (*p* = 0.636). Similar to previous studies, pre-*S* deletion and pre-*S*2 start codon mutation in pre-*S* gene, C1653T, and A1762T/G1764A mutations in *X* gene were associated with significantly higher risk of HCC development. Furthermore, in this full HBV genome comparison between 30 HCC cases and 30 controls, we also identify some rarely reported or new HCC-related mutations, including G1613A in the *X* gene, and A2159G, A2189Y, G2203W, and C2288R mutations in the *C* gene. These new high risk mutations—together with those confirmatory mutations in pre-*S* and Enhancer II/BCP regions from previous studies—suggested that mutation combinations in the full genome sequence might serve as potential viral markers for predicting the development of HBV-related HCC.

Among the 10 HCC-related mutations acquired from the full genome analysis in stage 1, four (A2159G, A2189Y, G2203W, and C2288R mutations) were located in the *C* gene. Meanwhile, the carcinogenic risk of pre-*S* deletion and pre-*S*2 start codon mutation in pre-*S* gene, C1653T, and A1762T/G1764A mutations in *X* gene has been extensively investigated in this cohort [17,18]. Compared to those studies focusing on mutations in pre-*S* and *X* genes, only a few studies have investigated the effect of mutations in the *C* gene during natural HBV infection. The clinical effect of mutations in this region was less well elucidated, and the results were inconsistent [19,20,21,22]. Thus, the temporal relationship between the *C* gene mutations and HCC in chronic HBV infection need to be further studied. In view of this, we carried out an independent validation study to confirm the findings from the initial full-length sequence comparison. After adjustment for age, history of cigarette smoking and alcohol consumption, unconditional logistic regression analyses showed that pre-*S* deletion, C1653T, A1762T/G1764A, A2159G, A2189Y, G2203W, and C2288R mutations were significantly associated with high HCC risk. Multivariate analysis indicated that pre-*S* deletion, A1762T/G1764A, A2159G, and A2189Y mutations were independent risk factors for HCC progression. To our knowledge, the clinical implications of these mutations in the *C* gene during HCC occurrence have been reported in very limited studies. Ni et al. reported that HCC children had more mutations in the *C* gene than chronic HBV carriers. The mutation sites at core codon 74, 87, and 159 were related to the development of HCC in a small scale study [21]. In a nested case–control study within a prospective study form Taiwan, six mutations in the *C* gene (nt 1961, 1938, 2045, 2136, 2239, and 2441) were identified to be associated with decreased risk of HCC after accounting for viral genotype. Meanwhile, these mutations were also related with a 0.7- to 1-log decrease in plasma viral load and a high rate of HBeAg sero-conversion [19]. However, we did not observe this protective effect of such mutations in the *C* gene on HCC development in the current study from Qidong in the mainland of China. We speculated that this is probably because of distinct clinical and virologic characteristics of HBV infection in different geographical parts of the world, such as the different prevalent HBV genotype and sub-genotype between Taiwan and Qidong. Recently, Zhu et al. demonstrated that A2189C and G2203W mutations were independent risk factors for HCC in another study from Qidong, showing odds ratios 3.99 and 9.70, respectively [22]. In accordance with the results of Zhu et al., in the present study, we confirmed that A2159G, A2189Y, G2203W, and C2288R mutations were associated with high HCC risk in univariate analysis. However, in multivariate analysis, G2203W and C2288R mutations were not independent risk factors in predicting HCC occurrence. The exact mechanism of hepatocarcinogenesis relating to the above *C* gene mutations remains uncertain. Theoretically, hepatitis B core antigen (HBcAg) contains the principal target for the cytotoxic T lymphocyte (CTL) attack and various epitopes of HBcAg recognized by immune cells, such as T or B lymphocytes. The amino acid substitutions in the *C* gene may permit a change in the immune recognition sites of HBcAg, thereby allowing the virus to elicit or evade immune clearance and have a more direct impact on the natural course of hepatitis B [23,24,25]. A2159G and A2189Y mutations are missense mutations resulting in an amino acid change of HBcAg codons 87 (S87G) and 97 (I97L/F), respectively. Because codon 87 is located in the B cell epitope of *C* gene, the missense substitution at codon 87 may alter the recognition site for B cells or antibodies and allow the virus to escape from attacking antibodies [26]. Substitution at codon 97 was the most frequently detected substitution of the *C* gene in this study. Since codon 97 is located within a potent T-cell epitope, this substitution may lower the quantity of antigen presentation in secretions of immature HBV particles. Thus, the codon 97 substitution may inhibit the immune response and lead to successful maintenance of chronic infection in human HBV carriers [27,28]. The prolonged viral persistence causes continuous liver injury and subsequent regeneration, which significantly increases the risk for HCC.

The effect of combined mutations in Enh II/BCP regions on increased risk of HCC was extensively identified in several studies [29,30,31]. It has been reported that most *C* gene mutations were prone to be clustered in the middle core region [32,33]. However, the majority of earlier studies primarily focused on the relationship between a certain point mutation of the *C* gene and HCC. In the present study, the risk of combined mutations in the *C* gene, including the A2159G, A2189Y, G2203W, and C2288R mutations, on HCC was explored. To examine the potential value of the presence of HBV mutation patterns—either alone or in combination—we evaluated the potential value of each mutation or combined mutations in the *C* gene for the prediction of HCC. The key finding of this study was that the number and pattern of multiple mutations in the *C* gene (A2159G, A2189Y, G2203W, and C2288R mutations) showed the additive combined effects that related to HCC progression. Compared to patients with wild-type in four hot spot nucleotides, our data indicated that the presence of any mutation combination in the *C* gene was associated with an increased risk of HCC. The OR of HCC cases that had any single hot spot mutation was 2.904 (95% CI, 1.508–5.590), it increased to 6.027 (95% CI, 2.232–16.275) with double mutations, and to 8.630 (95% CI, 1.588–46.891) with triple mutations. A significant biological gradient of HCC risk by an increasing number of mutations in the *C* gene was observed. We then recruited a series of serum samples spanning years before and after HCC diagnosis. The longitudinal observation demonstrated a sequential and accumulative combination of mutations in the *C* gene during the development of HCC. During the course of chronic HBV infection, it is speculated that the accumulation of HBV complex mutations may have a sequential and synergistic role in the development of HCC. Although the mechanism is unclear, this finding suggests that the detection of these combined mutations may aid in screening the high HCC risk subjects in chronic HBV carriers. Additionally, HCC mostly develops in patients with cirrhosis. Therefore, HCC and cirrhosis may share the same risk factors, including high HBV DNA levels, certain genotypes, and naturally occurring viral mutations. In view of this, we speculate that such mutations in the *C* gene were accompanied by the progression of advanced liver disease—not only HCC but also liver cirrhosis.

There are also some limitations that should be considered in the present study. First, most analysis of HBV mutations was based on a single blood sample obtained at the diagnosis of HCC, and we could not assess the effect of changes in mutation status on the development of HCC; Second, the direct sequencing method only revealed the predominant strains in the host, and it may underestimate the real mutation level in patients, as in most cases, mixture infection of different viral strains was common; Third, as an important risk factor of HCC, liver cirrhosis or fibrosis was not evaluated in this study; Finally, the result was limited because all the study subjects were males, a larger cohort and a longer follow-up time are needed for a similar study in females. Because there were several limitations existing in the current study, our results should be interpreted with caution. Likewise, the conclusions of this study should also be drawn cautiously. Therefore, future studies or analyses assessing the risk of *C* gene mutations on the development of HCC should be performed on the basis of overcoming such limitations.

## 4. Experimental Section

### 4.1. Study Population

This study was based on a prospective cohort in Qidong County, Jiangsu Province, China. The recruitment of this cohort has been described elsewhere [17,18]. Briefly, a total of 2387 males living in 17 townships of Qidong who were seropositive for hepatitis B surface antigen (HBsAg) and free of HCC at recruitment were followed up from August 1996 to October 2006. Study participants were scheduled to receive serum α-fetoprotein (AFP) level, conventional liver function, and ultrasonography measurements every 6 to 12 months. The diagnosis of HCC was based on the following criteria: 1 imaging technique and a serum AFP level ≥400 ng/mL; a histopathological examination; or a positive lesion detected by at least two different imaging techniques (US, CT, MRI, and hepatic angiography). Several HCC cases qualified based on more than one criterion. At entry of the program, each study participant provided written informed consent and a structured questionnaire to obtain information on demographic characteristics and lifetime habits of alcohol and tobacco consumption. Serum samples collected at interview were stored at −70 °C before analysis. The study was approved by the Clinical Research Ethics Committee of Affiliated Hospital of Nantong University, Jiangsu Province, China (date of approval, 18 May 1996; permission code, 1996025). The study was conducted according to the tenets of the Declaration of Helsinki.

### 4.2. Cases and Controls

The HCC data were obtained from medical records and searches of computer files of death certification and cancer registry systems. To ensure complete ascertainment, we also contacted relatives to identify cases. For the complete HBV genome analysis, 30 HCC cases and 30 non-HCC control patients were enrolled. In further validation of mutations in the C gene, we recruited an independent set of 100 HCC patients and 100 chronic hepatitis (CH) patients as controls. We have excluded the HCC cases that were diagnosed within the first two years of follow-up. Meanwhile, the controls from the cohort of HBsAg carriers were all alive and had not been diagnosed with HCC throughout the follow-up period from August 1996 to October 2006. The control patients were individually matched to the HCC cases by age (within two years). No subjects receiving anti-viral therapy were included. All 260 participants were positive for serum HBsAg and HBV DNA. For the longitudinal investigation, serial serum samples of 11 HCC patients were analyzed. Subjects were excluded if they had poor sequence data in the C gene (one case and two controls in the validation set). Consequently, a total of 129 cases and 128 controls were included in the analysis.

### 4.3. Serology

Serum HBsAg, HBeAg, and anti-HCV antibody were tested by commercially available enzyme immunoassay kits (Shanghai Kehua Bio-engineering Co., Ltd., Shanghai, China). Serum alanine aminotransferase (ALT) level was determined by ultraviolet-lactate dehydrogenase (UVLDH) method (Shanghai Kehua Bio-engineering Co., Ltd). The serum HBV DNA levels were determined using the Fluorescein quantitative polymerase chain reaction (FQ-PCR) detection system (Taqman; Roche Applied Science, Indianapolis, IN, USA), according to the manufacturer’s instructions. The lower limit of detection was 100 IU/mL.

### 4.4. Amplification and Sequencing of the Full HBV Genome and Pre-S/Enh II/BCP/C Regions

HBV DNA was extracted from 200 µL serum samples using a commercial Kit (Shanghai Shenyou Biotech Company, Shanghai, China). The HBV full-length sequence was amplified by PCR using FLP1 (5′-TTTTTCACCTCTGCCTAATCATCTCA-3′ (nt 1821–1846), sense) and FLP2 (5′-AAAGTTGCATGGTGCTGGTGAA-3′ (nt 1823–1802), antisense) as primers. PCR reaction was carried out in 50 µL containing 5 µL 10× buffer, 4 µL 2.5 mmol/L deoxynucleoside triphosphates (dNTP), 2 µL 10 µmol/L sense and antisense primers, and 1.5 U PlatinumTaq DNA polymerase (Invitrogen, Shanghai, China). PCR was performed as follows: 95 °C for 2 min; 95 °C for 30 s, 56 °C for 30 s, and 68 °C for 3 min for 35 cycles; and finally, 68 °C for 10 min. PCR products were purified (BioDev-Tech. Co., Ltd., Beijing, China) and cloned into the PMD18-T Vector (TaKaRa Bio, Dalian, China) for sequencing. Sequencing was conducted with ABI 3700 sequencer and a commercial kit (Applied Biosystems, Foster City, CA, USA) using PMD18-T vector universal primers and HBV-specific primers. For *C* region sequence analysis, *C* gene corresponding to nucleotides 1901 to 2450 was amplified by the nested PCR. First-round PCR primers were (5′-TTCACCTCTGCCTAATCATCTC-3′ (nt 1824–1845), sense) and (5′-TCTCCTGTTTTCATTTACTGTAA-3′ (nt 2624–2602), antisense). Second round PCR primers were (5′-TCCAAGCTGTGCCTTGGGTG-3′ (nt 1871–1890), sense) and (5’-GAAGAATAAAGCCCAGTAAA-3′ (nt 2500–2481), antisense). PCR was done under the same PCR condition described above, except the primers were used. Nested PCR for the amplification of pre-*S*, enhancer II, and basal core promoter regions was performed as previously described [17,18]. All necessary precautions to prevent cross-contamination were taken, and negative controls were included in each assay. Amplified products were directly sequenced in both the forward and reverse directions using an ABI 3700 sequencer and commercial kit (Applied Biosystems). Sequences of the complete genome or C gene and deduced amino acid sequences were aligned and compared by using the software MEGA version 4.1 (Biodesign Ins., Phoenix, AZ, USA).

### 4.5. HBV Genotyping

HBV genotyping was determined by phylogenetic analysis using the sequences of the HBV *C* gene. The compared standard genome sequences were The genetic distances were estimated by Kimura’s two-parameter method, and the phylogenetic trees were constructed by the neighbor-joining method. To confirm the reliability of downloaded from GenBank. HBV nucleotide sequences were multiple-aligned by the Clustal X program (MEGA version 4.1 software). The phylogenetic tree analysis, bootstrap re-sampling and reconstruction with 1000 replicates were used. These analyses were carried out using MEGA version 4.1 software.

### 4.6. Statistical Analysis

Data are presented as means ± SD, proportions, or median (range). To compare the values between the two groups, Pearson’s χ^2^ or Fisher exact tests were performed for categorical variables, and the Student’s *t*-test was used for continuous variables with normal distributions. Binary unconditional logistic regression models were used to estimate the odds ratios (ORs) of HCC associated with HBV-related factors and corresponding 95% confidence intervals (CIs). Potential confounders including age, and history of cigarette smoking and alcohol consumption were adjusted. Multivariate analyses with stepwise logistic regression were used to determine the independent factors correlated with HCC risk. All of the statistical tests were two-tailed, and *p* < 0.05 was considered statistically significant. All statistical analyses were performed using SPSS 11.5 for Windows (SPSS Inc., Chicago, IL, USA).

## 5. Conclusions

In summary, the pilot full genome analysis of HBV provided initial information for the identification of potential mutations in the *C* gene related to HCC. Then, an independent case–control study revealed that the A2159G and A2189Y mutations were independent risk factors for HCC in chronic HBV-infected subjects in Qidong. A combined examination of these mutations might help to predict the clinical outcomes of chronic HBV carriers more precisely, thus helping those who are at high-risk of HCC to benefit from early diagnoses and interventions.

## Figures and Tables

**Table 1:** Number of nucleotide substitutions in the full genome and various regions of the hepatitis B virus (HBV) genome in hepatocellular carcinoma (HCC) cases and controls.

Variable	Full Genome (nts 1–3215)	Pre-*S*1 (nts 2848–3204)	Pre-*S*2 (nts 3205–154)	*S* (nts 155–835)	*X* (nts 1374–1838)	Pre-*C*/*C* (nts 1814–2452)	*P* (nts 2307–1623)
HCC (*n* = 30)	66.3 ± 10.4	6.7 ± 1.5	4.5 ± 1.6	5.3 ± 1.3	9.1 ± 2.7	10.1 ± 2.4	30.5 ± 9.2
Controls (*n* = 30)	57.6 ± 7.8	6.4 ± 1.8	3.5 ± 1.5	5.2 ± 1.4	7.0 ± 2.4	8.4 ± 2.7	27.1 ± 7.1
*p*-value	0.001	0.483	0.015	0.636	0.002	0.016	0.112

**Table 2:** The prevalence of hot-spot mutations throughout the complete HBV genome.

Mutation	Amino Acid	Total *n* = 60 (%)	HCC *n* = 30 (%)	Controls *n* = 30 (%)	*p*-Value
Pre-*S* deletion	-	13 (21.7)	10 (33.3)	3 (10.0)	0.028
Pre-*S*1 gene					
C3026W	A 60 V/E	10 (16.7)	6 (20.0)	4 (13.3)	0.451
Pre-*S*2 gene					
Pre-*S*2 start codon	M 1 V/T/I	7 (11.7)	6 (20.0)	1 (3.3)	0.039
T31C	synonymous	16 (6.7)	5 (16.7)	11 (36.7)	0.128
T53C	F 22 L	14 (23.3)	10 (33.3)	4 (13.3)	0.056
*S* gene					
T531S	I 126 T/S	8 (13.3)	5 (16.7)	3 (10.0)	0.448
*P* gene					
G1053A	synonymous	11 (18.3)	7 (23.3)	4 (13.3)	0.317
G1229A	R713Q	21 (35)	13 (43.3)	8 (26.7)	0.176
*X* gene					
G1613A	synonymous	19 (31.7)	14 (46.7)	5 (16.7)	0.012
C1653T	H94Y	18 (30.0)	13 (43.3)	5 (16.7)	0.024
T1753V	I127T/N/S	12 (20.0)	8 (26.7)	4 (13.3)	0.197
A1762T	K130M	31 (51.7)	21 (70.0)	10 (33.3	0.004
G1764A	V131I	34 (56.7	23 (76.7	11 (36.7)	0.002
C1766T	synonymous	9 (15.0)	6 (20.0)	3 (10.0)	0.278
T1768A	F132Y	7 (11.7)	5 (16.7)	2 (6.7)	0.228
Pre-*C* gene					
G1896A	W28Stop	23 (38.3)	12 (40.0)	11 (36.7)	0.791
G1899A	G29D	8 (13.3)	5 (16.7)	3 (10.0)	0.448
*C* gene					
T1938C	V13A	6 (10.0)	5 (16.7)	1 (3.33)	0.085
C2002T	synonymous	5 (8.33)	4 (13.3)	1 (3.33)	0.161
T2045A	S49T	7 (11.7)	2 (6.67)	5 (16.7)	0.228
A2159G	S87G	19 (31.7)	14 (46.7)	5 (16.7)	0.012
A2189Y	I97L/F	20 (33.3)	15 (50.0)	5 (16.7)	0.006
G2203W	synonymous	5 (8.33)	5 (16.7)	0 (0)	0.020
C2288R	P130T/A	10 (16.7)	8 (26.7)	2 (6.67)	0.038

Abbreviations: W: A or T; S: G or C; R: A or G; Y: C or T.

**Table 3:** Clinical and virologic characteristics of 99 HCC cases and 98 controls.

Variables	HCC *n* = 99 (%)	Controls *n* = 98 (%)	Adjusted Odds Ratio * (95% CI)	*p*-Value
Age (year)	54.8 ± 8.4	54.0 ± 9.6	-	0.577
Cigarette smoking	52 (52.5)	46 (46.9)	-	0.433
Alcohol consumption	61 (61.6)	56 (57.1)	-	0.523
ALT > 45 U/L	24 (24.2)	21 (21.4)	1.196 (0.611–2.342)	0.602
HBeAg positive	35 (35.4)	24 (24.5)	1.676 (0.900–3.121)	0.104
HBV DNA levels (log_10_ copies/mL)	4.5 ± 1.2	4.3 ± 1.4	1.152 (0.925–1.435)	0.208
Genotype C	92 (92.9)	93 (94.9)	0.750 (0.222–2.531)	0.642
Pre-*S* deletion	25 (25.3)	12 (12.2)	2.385 (1.117–5.094)	0.025
Pre-*S*2 start codon	13 (13.1)	6 (6.1)	2.233 (0.795–6.274)	0.127
G1613A	35 (35.4)	23 (23.5)	1.769 (0.932–3.358)	0.081
C1653T	32 (32.3)	20 (20.4)	1.929 (1.003–3.707)	0.049
A1762T/G1764A	69 (69.7)	50 (51.0)	2.247 (1.230–4.106)	0.008
Deletion in *C* gene	6 (6.1)	2 (2.0)	3.556 (0.677–18.663)	0.134
T1938C	15 (15.2)	7 (7.1)	2.291 (0.873–6.013)	0.092
C2002T	11 (11.1)	7 (7.1)	1.707 (0.624–4.669)	0.298
T2045A	13 (13.1)	14 (14.3)	0.965 (0.421–2.211)	0.933
A2159G	35 (35.4)	20 (20.4)	2.147 (1.126–4.094)	0.020
A2189Y	40 (40.4)	22 (22.4)	2.403 (1.281–4.505)	0.006
G2203W	10 (10.1)	2 (2.0)	5.203 (1.103–24.541)	0.037
C2288R	22 (22.2)	11 (11.2)	2.437 (1.092–5.438)	0.030

Abbreviations: ALT, alanine aminotransferase; HBeAg, hepatitis B e antigen; W: A or T; S: G or C; R: A or G; Y: C or T; * Adjusted for age, history of cigarette smoking, and history of alcohol consumption.

**Table 4:** Multivariate analysis of independent factors for the risk of HCC.

Variables	Odds Ratio (95% CI)	*p*-Value
A2159G mutation	2.037 (1.033–4.015)	0.040
A2189Y mutation	2.833 (1.453–5.526)	0.002
Pre-*S* deletion	2.272 (1.037–4.979)	0.040
A1762T/G1764A mutations	2.333 (1.240–4.390)	0.009

**Table 5:** Association between HCC and the presence of specific mutation patterns in the *C* gene.

Type	Pattern	Total *n* = 197 (%)	HCC *n* = 99 (%)	Controls *n* = 98 (%)	Adjusted Odds Ratio * (95% CI)	*p*-Value
	A2159G A2189Y G2203W C2288R					
Wild type	− − − −	80 (40.6)	26 (26.3)	54 (55.1)	1.00 (reference)	
Single mutation		81 (41.1)	46 (46.5)	35 (35.7)	2.904 (1.508–5.590)	<0.001
	− + − −	33 (16.8)	17 (17.2)	16 (16.3)		
	+ − − −	22 (11.2)	19 (19.2)	13 (13.3)		
	− − − +	15 (7.6)	10 (10.1)	5 (5.1)		
	− − + −	1 (0.5)	0 (0)	1 (1.0)		
Double mutations		27 (13.7)	20 (20.2)	7 (7.1)	6.027 (2.232–16.275)	<0.001
	+ + − −	9 (4.6)	7 (7.1)	2 (2.0)		
	− + − +	6 (3.0)	4 (4.0)	2 (2.0)		
	+ − − +	6 (3.0)	4 (4.0)	2 (2.0)		
	− + + −	5 (2.5)	5 (5.1)	0 (0)		
	+ − + −	1 (0.5)	0 (0)	1 (1.0)		
Triple mutations		9 (4.6)	7 (7.1)	2 (2.0)	8.630 (1.588–46.891)	0.013
	+ + − +	4 (2.0)	2 (2.0)	2 (2.0)		
	+ + + −	3 (1.5)	3 (3.0)	0 (0)		
	− + + +	2 (1.0)	2 (2.0)	0 (0)		

* Adjusted for age, history of cigarette smoking, and history of alcohol consumption. Because of rounding, percentages do not always total 100. “−“, absence; “+”, presence.

**Table 6:** Longitudinal observation of *C* gene mutation patterns in 11 HCC cases.

Case	Age	Genotype	HBeAg	At Baseline	2–4 Years Before HCC	HCC
#5	45	C	−	○―●―●―○	○―●―●―○	●―●―●―○
#12	51	C	+	○―○―○―●	○―○―○―●	○―●―○―●
#13	55	C	−	○―○―○―○	○―○―○―○	○―○―○―○
#21	60	C	−	○―●―○―○	○―●―○―○	○―●―○―○
#45	45	C	+	Negative PCR product	○―○―○―○	○―○―○―○
#62	52	C	−	○―○―○―●	○―○―○―●	○―○―○―○
#65	49	C	−	○―○―○―○	●―○―○―○	●―○―○―○
#71	38	B	+	●―○―○―●	●―●―○―●	●―●―○―●
#87	56	C	−	○―○―○―○	Negative PCR product	○―○―○―○
#94	54	C	−	○―○―○―○	○―●―○―○	○―●―○―○
#98	49	C	−	○―○―●―○	○―○―●―○	○―○―●―○

○―○―○―○, 2159―2189―2203―2288; ○, wild type; ●, mutation type.

## Abbreviations

HBV	hepatitis B virus
HCC	hepatocellular carcinoma
BCP	basal core promoter
HBsAg	hepatitis B surface antigen
AFP	alpha-fetoprotein
CH	chronic hepatitis
FQ-PCR	fluorescein quantitative polymerase chain reaction
ORF	open reading frame
HBeAg	hepatitis B e antigen
HBcAg	hepatitis B core antigen
CTL	cytotoxic T lymphocyte
UVLDH	ultraviolet-lactate dehydrogenase
